# MicroRNA-1179 suppresses the proliferation and enhances vincristine sensitivity of oral cancer cells via induction of apoptosis and modulation of MEK/ERK and PI3K/AKT signalling pathways

**DOI:** 10.1186/s13568-020-01082-8

**Published:** 2020-08-18

**Authors:** Yanmei Gao, Hanmei Xu, Tiemin Pu

**Affiliations:** Department of Stomatology, Affiliated Hospital of Jilin Medical University, NO 81 of Road Huashan, Fengman Area, Jilin, 132013 China

**Keywords:** Oral cancer, Micro RNA, Proliferation, Autophagy, Metastasis

## Abstract

The role of miR-1179 in the development of cancer has been proved by different studies. However, the expression profile and role of miR-1179 is yet to be explored in human oral cancer. Consistently, this study was undertaken to explore the molecular role of miR-1179 in regulation of the human oral cancer development and progression. The results showed miR-1179 to be significantly (*p* < 0.05) overexpressed in all the oral cancer cell lines relative to normal cells. The repression of miR-1179 transcript levels not only suppressed the proliferation of oral cancer cells but also increased their sensitivity to vincristine. The decline in proliferative rates was attributed to induction of autophagy in oral cancer cells as confirmed by transmission electron microscopic analysis. Western blot analysis showed that the expression of LC3B-II increased and that of beclin 1 decreased while LC3B-I expression remained constant upon miR-1179 inhibition. Inhibition of miR-1179 caused significant decrease in the migration and invasion of the oral cancer cells. The migration and invasion found to be 47% and 32% for SCC-9 and 24% and 28% for SCC-25 cells upon miR-1179 inhibition. At molecular level, the miR-1179 was shown to exert its anticancer effects via deactivation of MEK/ERK and PI3K/AKT signalling cascades. In conclusion, the findings point towards the potential of miR-1179 in the treatment of oral cancer.

## Introduction

Despite advancement in science and technology, the current strategies employed for the treatment of human oral cancer are still far from descent. As such, presently the oral cancer is still ranked 6th most prevalent type of cancer, globally (Peers et al. 2019). The survival rates of oral cancer are comparatively lower than breast and prostate cancers. The overall 5-year survival rate of oral cancer is only 62% as against 89% and 99% for breast and oral cancers, respectively (Gupta et al. 2016). Additionally, the recurrence of human oral cancer further adds to the problem (Borsetto et al. 2019). Hence, it is need of the hour to look for the alternative approaches for the management of oral cancer. As revealed by the recent research reports, the molecular regulators of human cancers have emerged as vital targets to be studied for their usage against these deadly ailments (Cao et al. 2019). Among these molecular agents, the small RNA entities called microRNAs (miRs) which do not code for proteins but have molecular regulatory roles, have been shown to be involved in the development and tumorigenesis of number of human cancers including the human oral cancer (Gaezon et al. 2009, Wu et al. 2011). The miRs have not only been shown to regulate the proliferation of human cancers but have also been shown to have influence the metastasis to neighbouring tissues (Peng et al. 2011). MicroRNA-1179 (miR-1179) has been implicated in the regulation of proliferation and metastasis of different human cancers (Jiang et al. 2015, Krutovskikh et al. 2010, Song et al. 2018, Lin et al. 2018). The miR-1179 has been shown to inhibit the growth of the glioblastoma cells by inducing cell cycle arrest (Xu et al. 2017). The inhibition of metastasis of hepatocellular carcinoma has been reported to be due to interaction of miR-365 with ZEB2 (Gao et al. 2019). In yet another study, miR-1179 targets HMGB1 to suppress the proliferation of gastric cancer cells (Li and Qin 2019). Nonetheless, the role and therapeutic implications of miR-1179 has not be reported. This study was therefore designed to investigate the role of miR-1179 in human oral cancer cells via modulation of the MEK/ERK and PI3K/AKT signalling pathways.

## Materials and methods

### Cell lines and culture conditions

The oral cancer cell lines (SCC-4, SCC-9, SCC-15 and SCC-25) along with normal (EBTr) cell line were procured from ATCC, USA. The culturing of cell lines was performed using Dulbeccoʼs modified Eagleʼs medium (DMEM) (Thermo Scientific). The cell lines were maintained in a CO_2_ incubator at 37 °C with 5% CO_2_ concentration and relative humidity of 98%.

### Transfection

To stably transfect the oral cancer cells with miR-NC and miR-inhibitor, Lipofectamine 2000 (Thermo Scientific) was used and the procedure was carried as per the manufacturer guidelines.

### Expression analysis

The RNeasy Mini Kit (Qiagen) and miScript Reverse Transcription Kit (Qiagen) were respectively used to isolate the RNA from cancer and normal cell lines and for cDNA synthesis. The SYBR Green mix (Thermo Scientific) was used for performing the expression analysis of miR-1179 through quantitative real-time polymerase chain reaction (qRT-PCR) on QuantStudio 3.0 real time PCR (Thermo Scientific). The cycling conditions were as follows: 95 °C for 20 s, followed by 40 cycles of 94 °C for 15 s, and 57 °C for 1 min. The expression values were quantified using 2^− ddCt^ method. The real primer sequences were miR-F: 5′- GCGGAAGCATTCTTTCAT-3′ and miR-R: 5′- CAAGGGCTCGACTCCTGT-3′.

### Cell viability assay

To assess the proliferation rates of the oral cancer cells transfected with miR-NC and miR-inhibitor for 24 h and administered with/without 5 µM vincristine, the cells were cultured in 96-well plate for 24 h, 48 h, 72 or 96 h at 37 °C. Following this, the 3-(4,5-dimethylthiazol-2-yl)-2,5-diphenyl tetrazolium bromide (MTT) reagent (Thermo Scientific) was added at final concentration of 5%. After 4 h, 150 µl dimethyl sulfoxide (DMSO) was added for dissolving the crystals of formazan. Absorbance at 570 nm was taken using spectrophotometer to determine the proliferation rates of oral cancer cells.

### Electron microscopy

For the assessment of induction of cell autophagy, the cancer cells transfected with miR-NC or miR-inhibitor for 24 h were examined using Transmission electron microscope (TEM). Trypsin treatment was used to make the collection of cancer cells which were then washed and then fixed with glutaraldehyde (2%) in phosphate buffer (0.1 m). The Osmium tetroxide (1%) was then used for post-fixing of cells. The cells were then treated with 95% ethanol and embedded in resin. The ultramicrotome was used for section cutting which was followed by electron microscopy.

### Transwell chamber assay

The migration and invasion of oral cancer cells transfected with miR-NC or miR-inhibitor were determined by using Transwell chamber without or with matrigel coating. Here, the cell suspension containing approximately 6000 cells was poured into the upper portion of transwell chamber and lower portion was supplemented with 700 µl DMEM medium with 10% fetal bovine serum (FBS). Following 48 h incubation at 37 °C and 5% CO_2_ concentration, the cancer cells from the surface of membrane’s upper side were swabbed away with cotton swabs while the cells sticking to lower surface of membrane were fixed with 70% ethanol and the stained with crystal violet (0.1%). The cells were then examined and photographs were taken using 100× light microscope.

### Western blotting

For the estimation of protein concentrations, the western blotting technique was used. Precisely, after transfection with miR-NC or miR-inhibitor, the oral cancer cells were cultured for 24 h at 37 °C. Centrifugation was used to collect the cells which were then washed using PBS buffer. This was followed by treatment with RIPA buffer containing 50 mM Tris-HCl (pH 7.4), 150 mM NaCl, 1% Triton X-100, 0.1% SDS, 5 mM EDTA, 30 mM Na2HPO4, 50 mM NaF, 0.5 mM NaVO4, 2 mM phenyl methylsulfonyl fluoride, and 10% protease cocktail inhibitor. The total protein concentrations were estimated using Bradford assay. Equal protein concentrations were loaded on the Sodium dodecyl sulfate polyacrylamide gel electrophoresis (SDS-PAGE) from each sample. The gel was blotted to PVDF membrane followed by treatment with primary antibodies (Santa Cruz Biotechnology, Inc., Dallas, TX, USA) overnight at 4 °C. The blots were washed in tris buffered saline (TBS), incubated with horseradish peroxidase-conjugated secondary antibody (Santa Cruz Biotechnology, Inc.) for 1 h at 4 °C, washed again three times with TBS and chemiluminescence was captured on hyperfilm following incubating the blots in enhanced chemiluminescence reagent.

### Statistical analysis

The experiments were performed in triplicate and expressed as mean ± standard deviation (SD). The Student’s t-test was performed through GraphPad Prism 7.0 software. The *p*-values < 0.05 were taken as statistically significant difference.

## Results

### miR-1179 is upregulated in oral cancer cells

**Fig. 1:**
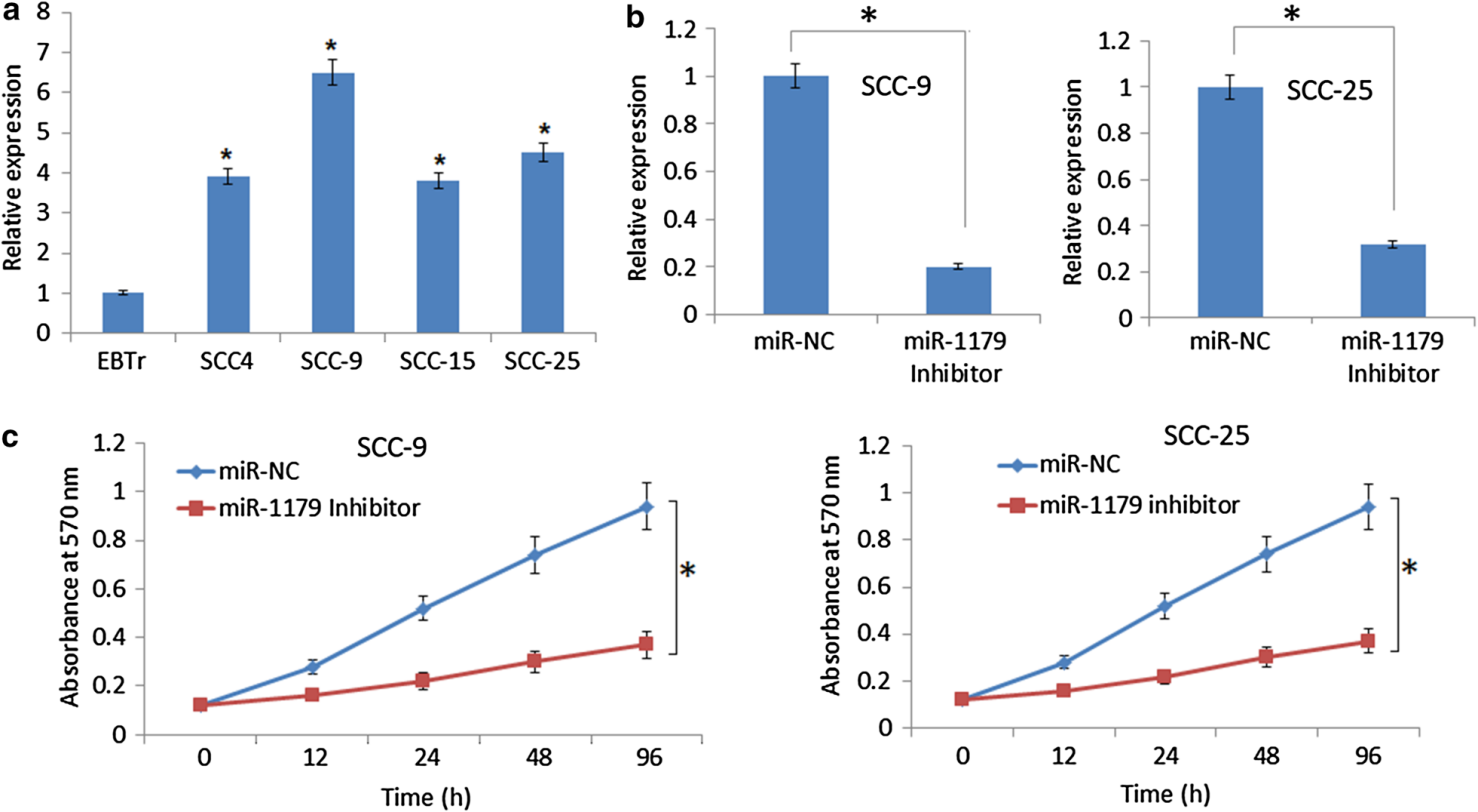
miR-1179 regulates the proliferation of human oral cancer cells **a** Expression of miR-1179 in human oral cancer and normal cells **b** Expression of miR-1179 in miR-NC and miR-1179 inhibitor transfected SCC-9 and ACC-25 cells **c** Cell viability of miR-NC and miR-1179 inhibitor transfected SCC-9 and ACC-25 cells. Individual experiments were performed in triplicate and expressed as mean ± SD (*P < 0.05)

The qRT-PCR analysis showed miR-1179 to be significantly upregulated in all the oral cancer cell lines studied (SCC-4, SCC-9, SCC-15 and SCC-25) relative to normal oral cell line (EBTr). The maximum transcript upregulation of miR-1179 was observed in SCC-9 oral cancer cells (6.5-fold) and SCC-25 cells (4.5-fold) relative to normal EBTr cells (Fig. 1a). As such SCC-9 and SCC-25 cell lines were taken for further experimentation.

### miR-1179 inhibition suppresses the proliferation and enhances chemosensitivity of oral cancer cells

**Fig. 2:**
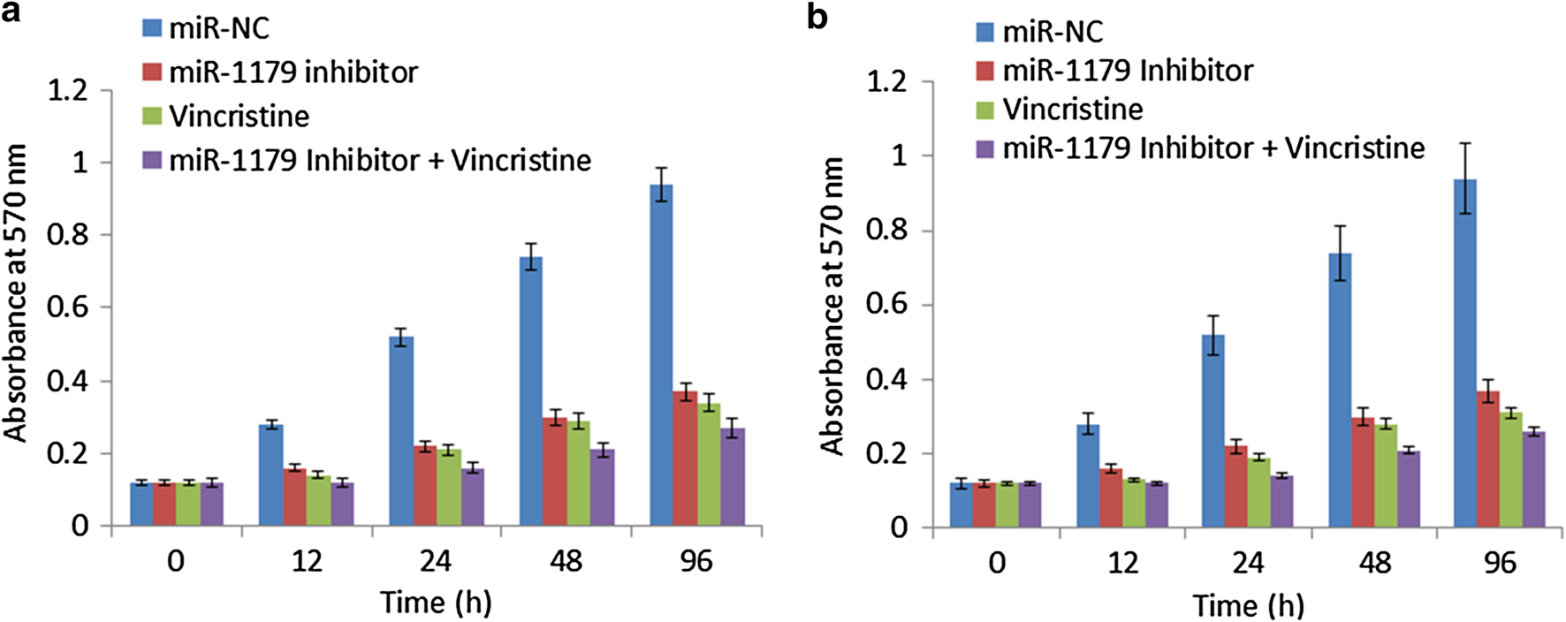
Inhibition of miR-1179 enhances the Vincristine sensitivity of the human SCC-9 and SCC-25 oral cancer cells. Individual experiments were performed in triplicate and expressed as mean ± SD (*P < 0.05)

To reveal the molecular functionality of miR-1179 in oral cancer, the suppression of miR-1179 was achieved by transfecting the SCC-9 and SCC-25 cancer cells with miR-1179 inhibitor. Using miR-NC transfected as control, the repression of miR-1179 transcript levels was confirmed by qRT-PCR method which showed significant (p < 0.05) decrease of miR-1179 expression in SCC-9 and SCC-25 cells (Fig. 1b) Additionally, the results showed that suppression of miR-1179 resulted in remarkable decline of proliferation rates of SCC-9 and SCC-25 cancer cells (Fig. 1c). Interestingly, the anti-proliferative effects of vincristine were shown to be greatly enhanced when SCC-9 and SCC-25 cancer cells were transfected with miR-inhibitor construct (Fig. 2). Together the results clearly indicate that miR-1179 inhibition suppresses proliferation and enhances the chemosensitivity of the human oral cancer cells.

### Inhibition of miR-1179 induces autophagy in the oral cancer cells

**Fig. 3:**
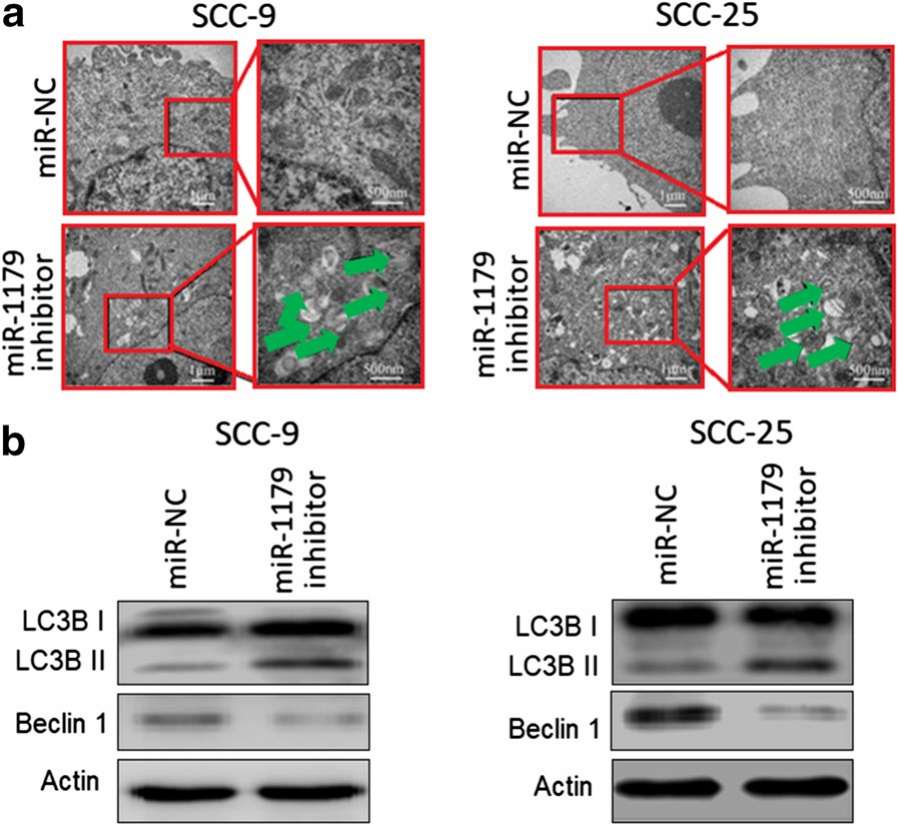
Inhibition of miR-1179 induces autophagy in oral cancer cells. **a** Ultrastructural analysis of the miR-NC and miR-1179 transfected SCC-9 and SC-25 cells. **b** Western blot analysis of miR-NC and miR-1179 transfected SCC-9 and SCC-25 cells showing the expression of LC3B I, II and Beclin 1. Individual experiments were performed in triplicate

The electron microscopic examination of SCC-9 and SCC-25 human oral cancer cells transfected with miR-1179 inhibitor or miR-NC clearly revealed the presence of autophagy vesicular structures in miR-inhibitor transfected cancer cells (Fig. 3a). However, such structures were totally lacking from the cancer cells transfected with miR-NC. The results were further supported by the western blotting of LC3B-I, LC3B-II and beclin 1 autophagy related proteins. The LC3B-I protein levels remined constant, beclin 1 protein levels declined and the LC3B-II protein levels increased in miR-1179 inhibitor transfected SCC-9 and SCC-25 oral cancer cells (Fig. 3b). Hence, it is evident that the transcript repression of miR-1179 in human oral cancer cells declines the cell proliferation rates via induction of cell autophagy.

### miR-1179 suppression reduces oral cancer cell metastasis

**Fig. 4:**
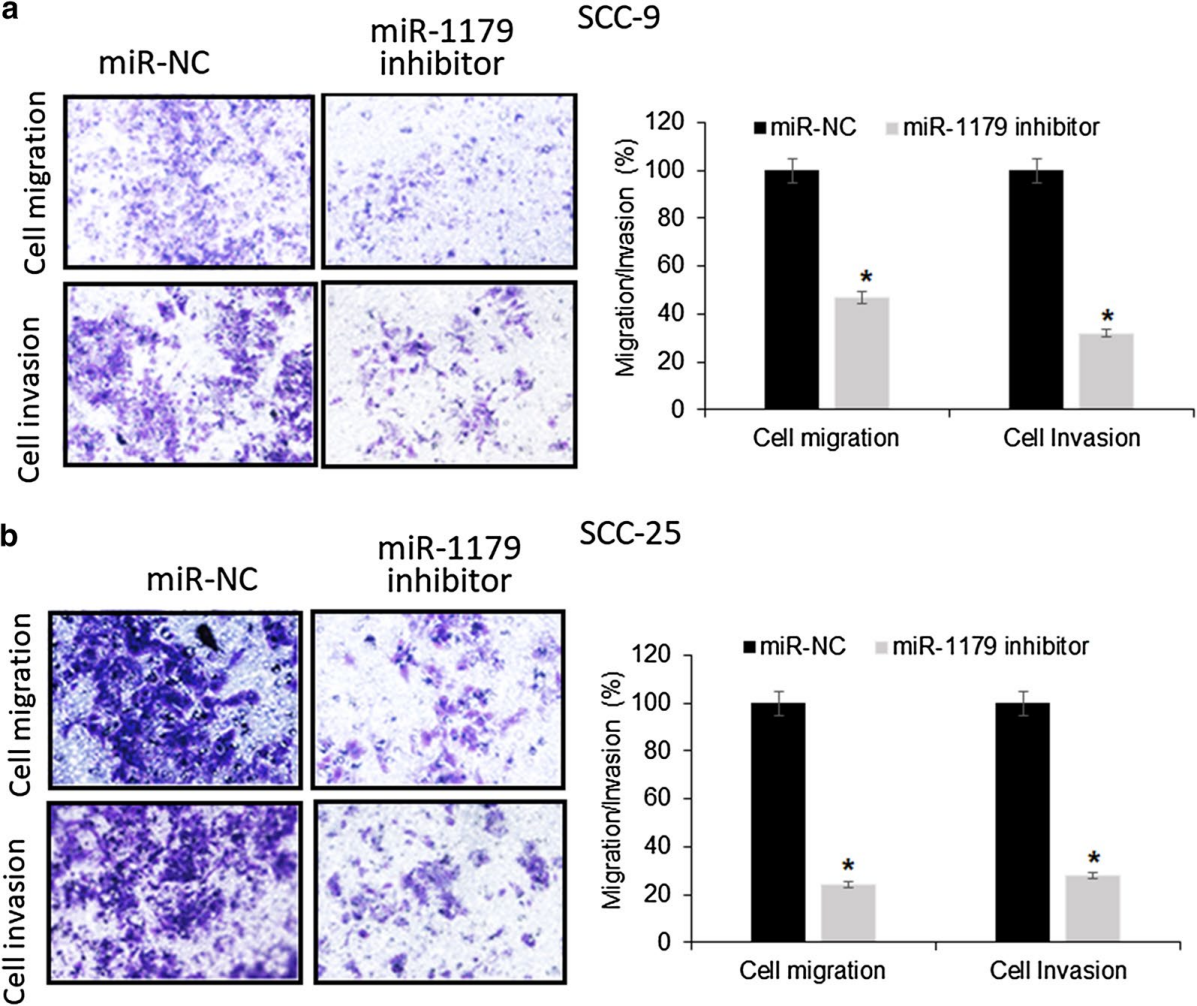
Inhibition of miR-1179 inhibits the migration and invasion of miR-NC and miR-1179 transfected SCC-9 and SC-25 cells. Individual experiments were performed in triplicate and expressed as mean ± SD (*P < 0.05)

The assessment of migration and invasion capabilities of SCC-9 and SCC-25 oral cancer cells transfected with miR- 1179 inhibitor or miR-NC constructs by transwell chamber assay showed that the miR-1179 inhibitor mediated suppression of miR-1179 expression resulted in significant decline in the migratory and invasive potential of SCC-9 and SCC-25 cancer cells (Fig. 4). The migration and invasion found to be 47% and 32% for SCC-9 and 24% and 28% for SCC-25 cells upon miR-1179 inhibition. Taken together, the results indicate that miR-1179 has a regulatory role on cancer cell metastasis.

### miR-1179 modulates MEK/ERK and PI3K/AKT pathways in oral cancer

**Fig. 5:**
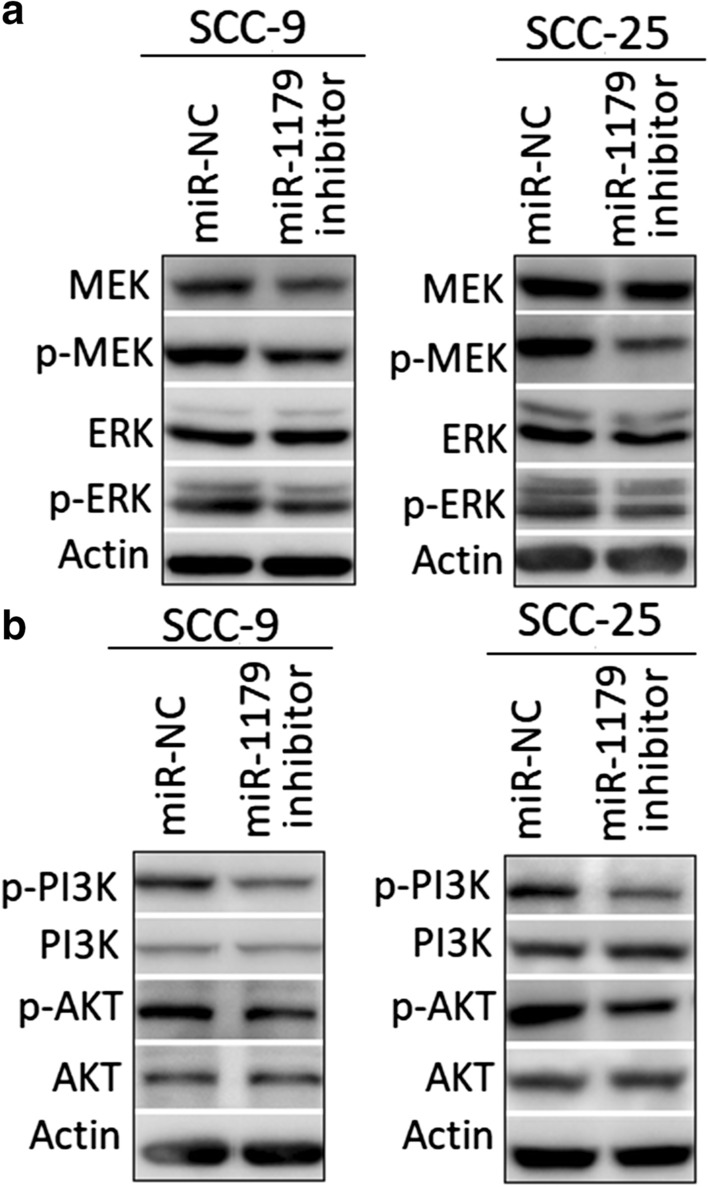
Inhibition of miR-1179 blocks MEK/ERK and PI3K and AKT signalling pathways. **a** Western blots showing the effects of miR-1179 inhibition on expression of MEK, p-MEK, ERK and p-ERK proteins in SCC-9 and SCC-25 cells. **b** Western blots showing the effects of miR-1179 inhibition on expression of PI3K, p-PI3K, AKT and p-AKT proteins in SCC-9 and SCC-25. The experiments were performed in triplicate

In order to analyse the molecular mechanics of miR-1179 in oral cancer, the repression of miR-1179 was made in SCC-9 and SCC-25 oral cancer cells. The assessment of MEK/ERK signalling pathway revealed that the decline in miR-1179 expression reduced the phosphorylated-MEK and ERK (p-MEK and p-ERK) protein levels (Fig. 5a). However, the protein levels of non-phosphorylated versions of MEK and ERK remained almost unchanged. Similarly, the reduction in phosphorylated protein versions of PI3K and Akt proteins (p-PI3K and p-Akt) was observed under miR-1179 repression (Fig. 5b). The protein level of PI3K and Akt remained unchanged. Taken together, the results reveal that the suppression of miR-1179 expression in oral cancer results in the blocking of phosphorylation of MEK, ERK, PI3K and Akt proteins.

## Discussion

Oral cancer is one of the lethal human disorders and this malignancy is responsible for a considerable level of human mortality and morbidity (Warnakulasuriya 2009, Peterson 2009). Overall, the oral cancer ranks 6th in terms of its incidence rates, globally. One more worrying fact about human oral cancer is its recurrence and development of drug resistance (Silva et al. 2012). So, an urgency is felt in the scientific community to devise more robust measures for the oral cancer management. Recently, miRs have emerged as potent regulators of various human cancers as they have been seen to have profound role in human physiology and disease development (Gong et al. 2005). The deregulation of miR-1179 has been implicated to be associated with a number of human cancers (Peng et al. 2011). In the present study, miR-1179 was found to be upregulated in oral cancer cells which is in accordance with several previous studies (Peng et al. 2011, Krutovskikh et al. 2010). Further, miR-1179 downregulation was previously been shown to decline the viability of ovarian cancer cells (Zhihong et al. 2019). Similar, observations were made from the results of the current study. The miR-1179 repression was found to inhibit proliferation and enhance the drug sensitivity of oral cancer cells to vincristine. Such implications have been drawn also previously (Zhihong et al. 2019). The characterization of miR-1179 in oral cancer in this study revealed that autophagy is induced in oral cancer cells when miR-1179 transcript levels are repressed. The autophagy was confirmed by the presence of autophagy vesicles which was further implicated by the enhancement of LC3-II conjugated protein expression level and declining of LC3-I and Beclin 1 expression. The miR-1179 repression in oral cancer cells further declined the migration and invasion rates of cancer cells which is in conformity with the previous studies on miR-1179 (Jiang et al. 2015). Lastly, among the most important molecular events of cancer progression, the activation of MEK/ERK and PI3K/AKT pathways hold a prime essence as these pathways enable the transcription of various downstream regulators of cancer growth and proliferation (Huang et al. 2018). These pathways have been shown to be aberrantly activated in different cancer types. For example, PI3K/AKT pathway has been shown to be activated in pancreatic cancer (Lan et al. 2019). In thyroid cancer cells, PI3K/AKT and MAPK/ERK pathway has been shown to significantly overexpressed (Su et al. 2019). Similarly, MEK/ERK pathway has shown to be activated in ovarian cancer (Zhang et al. 2019). As such, the blockage of these signalling pathways is a vital asset to keep the cancer progression at check (Zhihong et al. 2019). The miR-1179 suppression renders the MEK/ERK and PI3K/Akt pathways to proceed at fairly diminished rates by preventing the phosphorylation of crucial signalling components like MEK, ERK, PI3K and AKT. Summing up, the current study deduced the molecular functionality of miR-1179 and implicated its molecular targeting in the management of human oral cancer. To conclude, the present study showed miR-1179 to be significantly upregulated in human oral cancer cells. Inhibition of miR-1179 overexpression resulted in decline of proliferation and metastasis and enhancement of chemosensitivity of human oral cancer cells. Additionally, miR-1179 also resulted in the blockage of the MEK/ERK and PI3K/AKT signalling pathway, pointing towards the therapeutic implications of miR-1179 in oral cancer treatment.

## Data Availability

Not applicable.
